# The prolactin receptor gene (*PRLR*) is linked and associated with the risk of polycystic ovarian syndrome

**DOI:** 10.1186/s13048-023-01280-5

**Published:** 2023-11-22

**Authors:** Mutaz Amin, Claudia Gragnoli

**Affiliations:** 1 INSERM, US14-Orphanet, Paris, 75014 France; 2 Division of Endocrinology, Department of Medicine, Creighton University School of Medicine, Omaha, NE 68124 USA; 3 Department of Public Health Sciences, Penn State College of Medicine, Hershey, PA 17033 USA; 4 Molecular Biology Laboratory, Bios Biotech Multi-Diagnostic Health Center, Rome, 00197 Italy

**Keywords:** Prolactin, PRL, Prolactin receptor, *PRLR*, Gene, Expression, Polycystic ovarian syndrome, PCOS, Ovary, Cortisol, Hypothalamic-pituitary-ovarian axis, HPO-axis, Metabolic, Insulin resistance, IR, Obesity, Type 2 diabetes, T2D, Families, Familial, Peninsular, Italy, Italian, Parametric analysis, Linkage disequilibrium, Association, Single nucleotide polymorphisms, SNP, Risk, Variant, Model, Dominant, Recessive, Penetrance, Complete, Incomplete, Irregular menses, Oligomenorrhea, Subfertility, Folliculogenesis, Fat metabolism, Ethnic group, Appetite, Energy expenditure, Estrous cycle length, Follicle luteinization, Maturation, Metabolism, Adrenal, Steroidogenesis, Anovulation, Testosterone, Hyperandrogenism, Hyperandrogenemia, Endocrine, Disorder, Women, Reproductive age, Impaired glucose metabolism, Milk production, Social bonding, Genotyping, Mendelian, PLINK, Pseudomarker, Testosterone, Miscarriage, Recurrent, Depression, Rotterdam diagnostic criteria, Identical twins, Uncertain paternity, Generation, Endometrium, Obese, RegulomeDB, Control, rs13436213, rs1604428

## Abstract

The prolactin receptor gene (*PRLR*) may contribute to polycystic ovarian syndrome (PCOS) since it plays important roles in physiological ovarian functions. *PRLR*-knockout mice have irregular cycles and subfertility and variants in or around the *PRLR* gene were associated in humans with female testosterone levels and recurrent miscarriage. We tested 40 variants in the *PRLR* gene in 212 Italian families phenotyped by type 2 diabetes (T2D) and PCOS and found two intronic *PRLR*-variants (rs13436213 and rs1604428) significantly linked to and/or associated with the risk of PCOS. This is the first study to report *PRLR* as a novel risk gene in PCOS. Functional studies are needed to confirm these results.

## Introduction

Prolactin (PRL) is well-known for its role in milk production and social bonding and less known for its role in insulin secretion and normal ovarian function [1–3]. PRL was shown to play a role in ovarian follicular development and maintenance of the corpus luteum [2]. These effects are mediated by the prolactin receptor (PRLR) which is encoded by the *PRLR* gene and whose variants are implicated in glucose homoeostasis [1] and gestational diabetes [4]. Both the *PRL* and the *PRLR* are expressed locally in the ovaries of premenopausal women controlling follicular formation and maturation as well as ovulation [5], possibly through paracrine or autocrine action [6]. The *PRLR* gene can potentially be implicated in the risk of polycystic ovarian syndrome (PCOS), which is a common condition characterized by anovulation, hyperandrogenism, insulin resistance, and polycystic ovaries [7]. More than one-third of PCOS patients have abnormally high PRL levels [8]. And the expression of the *PRLR* gene is reduced in the endometrium of women with PCOS [9] and in mouse models with PCOS-like phenotype [10], reinforcing the link between the PRL system and PCOS. High PRL levels also impair the gonadotropin-releasing hormone (GnRH) release and pulsatility [11], which then affects GnRH effects on the release of the gonadotropins follicle-stimulating hormone (FSH) and luteinizing hormone (LH) [12], which respectively stimulate ovarian follicles formation and ovulation, thereby impairing the ovulatory cycles [13]. Furthermore, *PRLR*-knockout mice have irregular cycles and subfertility [14] and variants in or around the *PRLR* gene were associated with changes in female testosterone levels [15] and recurrent miscarriage [16]. Thus, an impaired PRLR function might mediate altered effects of PRL on GnRH and the hypothalamic-pituitary-ovarian axis, reproducing the PCOS abnormalities [6]. Yet, no study has reported *PRLR* as a risk gene in PCOS. In this study we report for the first-time novel risk variants in the *PRLR* gene significantly linked and associated with the risk of PCOS in Italian families.

## Materials and methods

We have tested 40 variants in the *PRLR* gene in 212 peninsular Italian families originally recruited for a type 2 diabetes (T2D) study and subsequently phenotyped for PCOS according to the PCOS Rotterdam diagnostic criteria (presence of at least two of these three characteristics: chronic anovulation or oligomenorrhea, clinical or biochemical hyperandrogenism, and/or polycystic ovaries) [17]. Identical twins and cases of uncertain paternity were excluded. The subjects were at least Italian for 3 generations [18].The samples were previously collected from the subjects’ whole blood and underwent the traditional phenol/chloroform DNA extraction method. Primers were chosen from the Affymetrix microarray database used that were available for the *PRLR* gene. The SNPs were selected based on their presence within the gene and validation in at least 3 public genetic databases. SNPs to be considered valid had to reach a quality control of at least 0.96. Random replicates from the samples were run to verify the accuracy of the results. PLINK [18] was used to detect genotyping or Mendelian errors allowing to identify any potential sample swap or paternity uncertainty or adoption case. The analyses we ran were free of any potential error. We used Pseudomarker [19] to test for 2-point parametric-linkage to and linkage disequilibrium (LD, that is linkage + association) with PCOS across the models: dominant with complete penetrance (D1), dominant with incomplete penetrance (D2), recessive with complete penetrance (R1), and recessive with incomplete penetrance (R2). Pseudomarker allows to test for linkage and LD (i.e., linkage + association) in extended pedigrees, affected siblings, trios (parents and affected child), and for association within unrelated individuals across families. Both linkage and LD under various hypotheses are tested. The LD/linkage can be considered the most important test, as it tests for both linkage and association, given the presence of linkage for the marker under analysis. Variants with *p* < 0.05 were considered significant. We performed functional bioinformatics analysis for the 2 PCOS-risk variants testing for disruption of transcription-factor binding (SNP2TFBS [20]), splicing (SNP-function prediction [21]) miRNA binding (mirSNP [22]), and/or regulation potential (RegulomDB [23]).

 [23]**Results and Discussion**.

We detected two intronic *PRLR*-variants (rs13436213 and rs1604428) significantly linked to/in LD with PCOS across the two recessive models (R1 and R2) (*p* < 0.05) (Table 1). Both variants are novel and have not been linked to any of PCOS-related phenotypes (i.e., obesity, insulin resistance, T2D, metabolic syndrome, hyperglycemia, hyperandrogenism, male-pattern baldness, acne, hirsutism, infertility, oligomenorrhea, anovulation or irregular menses). The same risk allele (C) of the variant (rs1604428) was significantly linked to the risk of depression in a previous analysis of the same dataset [24] Women with PCOS have increased risk of depression [25] and the *PRLR*-rs1604428 variant could partially explain this association. The functional bioinformatics analysis for the 2 PCOS-risk variants in our study found that the variants intersect with repressed chromatin state and potentially negative *PRLR* gene expression in the uterine tissue according to the analysis done by RegulomeDB [23] which predicts the regulatory potential of variants such as transcription-factor binding and chromatin state. Interestingly, this is consistent with a previous study reporting lower *PRLR*-mRNA levels in the endometrium of obese patients with PCOS compared to controls [9].

**Table 1:** Polycystic ovarian syndrome (PCOS) *PRLR*-risk single nucleotide polymorphisms (SNPs)

SNP	Position	Ref/Alt	Risk Allele	Consequence	Model^1^	*p* (LD|Linkage)	*p* (LD|NoLinkage	*p* (Linkage|LD)
rs13436213	35,185,724	C/T	C	Intronic	R1	0.08	0.04*	0.08
rs1604428	35,227,946	C/T	C	Intronic	R1	0.01*	0.03*	0.02*
					R2	0.04*	0.09	0.07

^1^Models: R1: recessive, complete penetrance, R2: recessive, incomplete penetrance. *Statistically significant

## Conclusion

This is the first study to report *PRLR* as a novel risk gene in PCOS. Functional studies are needed to confirm these results.

## Data Availability

The study data are available on reasonable request, and due to lacking specific patients’ consent and privacy restrictions, they are not publicly available.
